# C-peptide and epicardial adipose tissue in dialysis-dependent chronic kidney disease patients

**DOI:** 10.3389/fmed.2025.1650859

**Published:** 2025-09-04

**Authors:** Luis D’Marco, Ana Checa-Ros, Antonella Locascio, Owahabanun Joshua Okojie, Iris Viejo, Valmore Bermúdez, Cristina Karohl, Paolo Raggi

**Affiliations:** ^1^Grupo de Enfermedades Cardiorrenales y Metabólicas, Departamento de Medicina y Cirugía, Universidad Cardenal Herrera-CEU, CEU Universities, Valencia, Spain; ^2^Division of Cardiology, Department of Medicine, Alberta Heart Institute, University of Alberta, Edmonton, AB, Canada; ^3^Aston Institute of Health & Neurodevelopment, Aston University, The Aston Triangle, Birmingham, United Kingdom; ^4^Departamento de Ciencias Biomédicas, Universidad Cardenal Herrera-CEU, CEU Universities, Valencia, Spain; ^5^Nephrology Department, Hospital Universitari i Politècnic La Fe, Valencia, Spain; ^6^Facultad de Ciencias de la Salud, Universidad Simón Bolívar, Barranquilla, Colombia; ^7^Faculdade de Medicina, Universidade Federal do Rio Grande do Sul, Porto Alegre, Brazil

**Keywords:** C-peptide, epicardial adipose tissue, vascular calcification, chronic kidney disease, dialysis, ESKD

## Abstract

**Background:**

Several studies suggest that C-peptide (CP) is involved in regulating lipolysis, adipokine release, and other functions in the adipose tissue. On the other hand, organ-specific adipose tissues, such as the epicardial adipose tissue (EAT), have been reported as an independent cardiovascular risk factor in patients on dialysis. This study aimed to evaluate the association between CP, EAT volume, and coronary artery calcification (CAC) as markers of cardiovascular risk, on subjects with type 2 diabetes receiving insulin and dialysis.

**Methods:**

This is a retrospective study on 62 patients with chronic kidney disease (CKD) stage 5 on dialysis awaiting kidney transplantation and referred for cardiovascular risk stratification at the Emory University Hospital. Computed tomography (CT) was used to assess CAC and to measure EAT volume. Demographic and anthropometric data were collected from all patients through record review.

**Results:**

The mean patient age was 43 ± 11 and 55% were women. None of the serum analytical parameters correlated with CP. Subjects with higher BMI exhibited higher levels of CP. EAT volume strongly correlated with CP levels, and it was significantly correlated with CAC. On the contrary, no correlation was found between CP and CAC.

**Conclusion:**

The significant association between EAT volume and CP suggests a potential role of CP in the cardiovascular physiopathology of patients with ESKD on dialysis. Insufficient statistical power was probably the cause of the lack of association of CP with CAC. Observational prospective studies are required to characterize CP as a cardiovascular risk marker in patients with ESKD.

## Introduction

The deleterious effects of hyperinsulinemia, both endogenous and exogenous, manifest as weight gain and several metabolic and neurohumoral alterations leading to cardiorenal damage (1, 2).

C-peptide (CP), a peptide composed of 31 amino acids released by the pancreatic *β*-cells in the same equimolar ratio as insulin into the blood, is clinically used in monitoring pancreatic β-cell function and insulin needs in patients with diabetes (3). Recent studies suggest that this peptide may exhibit hormonal properties targeting various tissues (4), although there exists some controversy regarding its actions in physiological and under diverse pathological conditions. According to *in vitro* and *in vivo* studies, CP reduces apoptosis in the heart, aorta, kidney and vascular endothelial cells by downregulating the activation of transcription factor p53 (5, 6). Under physiological conditions, CP is also reported to play an antioxidant role by restoring the mitochondrial electron transport chain activity and thereby reducing the generation of reactive oxygen species (ROS) (7, 8). Additionally, CP seems to be involved in the secretion of adipokines and adipose tissue functioning (9, 10). However, CP oversecretion, as it occurs in pathological states such as obesity, is linked to the production of pro-inflammatory factors (IL-6, IL-1β), inhibition of lipolysis, increased secretion of the pro-inflammatory adipokine leptin (11, 12) and mesenchymal stem-cell dysfunction (13). In patients with diabetes, opposing effects from CP have been described for type-1 (T1DM) and type-2 diabetes mellitus (T2DM). On the one hand, higher levels of CP have been associated with a lower long-term complication rate in T1DM (14), including less glomerular hypertrophy, hyperfiltration, and proteinuria (15). On the other hand, CP has been reported to have pro-inflammatory and pro-atherogenic effects in T2DM patients by accumulating in the vessel wall (16–18) and inducing microvascular damage. Clinical research has uncovered that in obese patients suffering from T2DM, there exists a positive correlation between CP concentrations and pro-inflammatory chemokines (C-C chemokine ligand 2 (CCL2)), as well as E-selectin, among others.

Visceral adipose tissues, including the epicardial adipose tissue (EAT) located below the visceral pericardium, have been associated with an increased risk of cardiovascular events both in the general and dialysis-dependent populations (19–23). In patients with coronary artery disease, the EAT is inflamed and releases a large quantity of inflammatory cytokines and pro-atherogenic mediators (24). EAT has been proposed to promote atherosclerosis via paracrine and autocrine effects (25–27).

A positive correlation between CP and EAT in patients with T2DM and obesity have been reported by prior studies (28, 29), suggesting the potential involvement of CP in systemic inflammation and cardiovascular events. We hypothesized that increased CP levels in patients at high cardiovascular risk, such as those with end-stage chronic kidney disease (ESKD), are associated with EAT volume and coronary artery calcium (CAC), two known risk markers. If this were demonstrated, CP may become a useful tool to gauge the undesirable side effects of long-term insulin therapy.

Patients with ESKD undergoing dialysis represent a unique clinical population in terms of insulin and CP metabolism. Given that their renal function is markedly diminished, degradation and clearance of insulin and CP are considerably decreased, leading to their accumulation regardless of endogenous pancreatic secretion (30). Consequently, the measurement and interpretation of CP in this group require specific consideration, as standard reference ranges may differ from the general population. Furthermore, most of the patients with T2DM on dialysis receive exogenous insulin, which can further increase circulating insulin and CP levels, contributing to visceral adiposity (EAT) and microvascular damage (CAC).

Our study aims to evaluate the relationship between CP, EAT and CAC in patients with T2DM on dialysis to further understand the potential involvement of CP in the common cardiovascular complications occurring in this population.

## Methods

### Study population

This is a cross-sectional study on 62 patients with ESKD undergoing renal replacement therapy (RRT) who were awaiting kidney transplantation. All patients were referred for cardiovascular risk stratification at Emory University Hospital (Atlanta, GA, USA), between December 2009 and October 2012. The cohort included 43 patients receiving hemodialysis and 19 receiving peritoneal dialysis, all of them with a previous diagnosis of T2DM and on treatment with insulin.

This study received approval from the Emory University Institutional Review Board (approval number: 23/424) and was performed in accordance with the Declaration of Helsinki as revised in 2013. Adult participant consent was waived given the retrospective nature of the study. The study protocol has been already detailed in a prior publication (19), and it is briefly summarized here.

### Clinical data collection

Demographic, anthropometric and clinical data were collected from the electronic medical records by LDM (further curated by OJO, AL and IV). Patients who smoked regularly were classified as current smokers. Hypertension was defined as a blood pressure >140/90 mmHg, or as the current intake of antihypertensive medications. History of cardiovascular disease included known coronary artery disease, heart failure, stroke, and peripheral vascular disease.

### Analytical data collection

Blood samples to measure glucose, lipid and mineral metabolism, as well as renal function markers, were collected after 12 h of fasting and a 15-min resting period, and stored at a temperature between 4°C and 15°C. The samples were later centrifuged in cold and measured using standard assays at the Emory University Hospital. CP levels were determined on an advanced automated immunoassay analyzer (ADVIA Centaur, Siemens, Forchheim, Germany: reference range 0.05-30 ng/mL; CV ~ 6%).

### Imaging protocol

Cardiovascular risk assessment was performed using either positron emission tomography with computer tomography (PET-CT; Siemens Biograph, 40-slice CT, Siemens, Forchheim, Germany), or single photon emission tomography with CT (SPECT–CT; GE Discovery NM 530, GE Medical Systems, Milwaukee, WI, USA). Patients were instructed to fast and avoid caffeinated beverages or theophylline-containing medications for a minimum of 12 h prior to imaging. Initial scout and transmission CT scans were obtained for orientation and attenuation correction with patients breathing shallowly. A further non-contrast CT scan was obtained during breath-hold for the evaluation of vascular calcification and EAT volume analyses (as described below), using prospective ECG triggering at 3-mm slice thickness to minimize radiation exposure.

Laboratory and imaging assessments were carried out on the same day. Measurements of vascular calcifications and EAT volume were performed by experienced investigators (CK, PR) blinded to clinical and analytical variables.

#### Coronary artery calcium scoring

**Figure 1:**
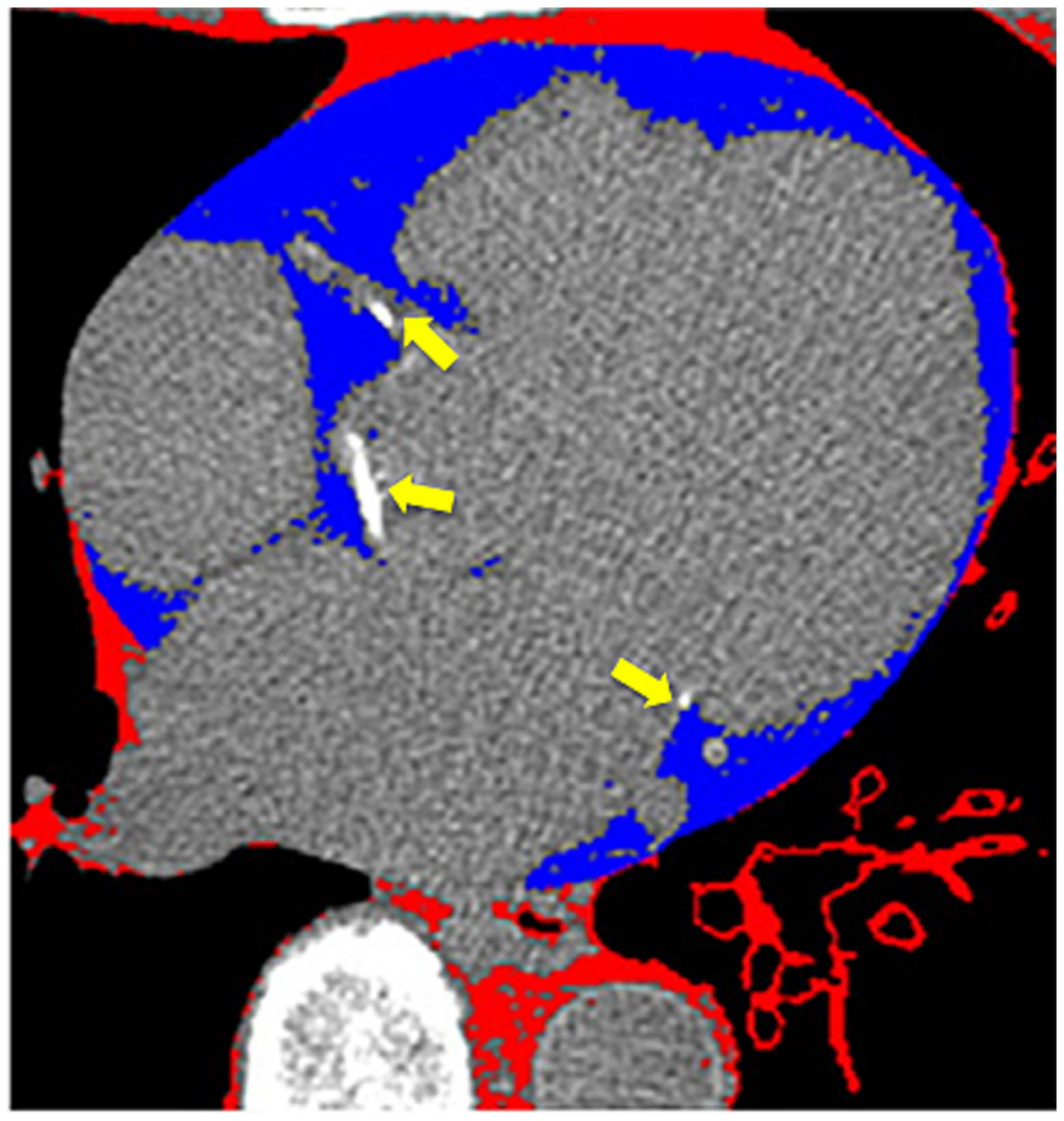
Cardiac computer tomography imaging. The arrows show calcium deposits in the coronary arteries. Blue-colored areas represent the epicardial adipose tissue.

Each area of calcification in the coronary arteries was scored using a semiautomatic software available on the workstation (Leonardo, Siemens Medical Solutions, Forchheim, Germany). CAC was considered present if a minimum of three contiguous pixels with an attenuation of ≥130 Hounsfield Units (HU) were detected along the course of a coronary artery (Figure 1, arrows). The Agatston score was used to quantify coronary artery calcium (CAC).

#### Epicardial adipose tissue measurement

EAT was identified as a hypodense region surrounding the myocardium and bounded by the pericardium on CT, using the Volume Software (Leonardo workstation, Siemens, Forchheim, Germany). The pericardium was manually traced from the mid-left atrium to the apex of the left ventricle, using a 10-mm thickness. By applying an attenuation threshold between-250 and-30 HU, many structures such as the myocardium, coronary arteries, coronary calcium, aorta, and blood pool were effectively excluded, highlighting only the EAT (Figure 1, blue area). Individual EAT volumes at each level were summed to calculate the total EAT volume. The estimated total radiation dose for the chest CT was 2.5 mSv.

### Statistical analysis

Normally distributed continuous variables were reported as mean ± standard deviation (SD). Categorical parameters were presented as absolute or relative frequencies (percentages). The Student’s t-test was used to compare normally distributed parameters. Categorical data were compared using the Chi-square test. Spearman’s bivariate correlations were conducted to test the strength of the association between variables.

Statistical analyses (VB, LDM, ACR) were performed using the statistical software package SPSS version 19 (SPSS Inc., IBM). A two-tailed *p-*value <0.05 was considered statistically significant.

## Results

### Demographics and clinical characteristics

The demographic, clinical, anthropometric, and analytical characteristics of the patients enrolled in this study are summarized in Table 1. Mean age of patients was 43 ± 11 years, with a slight female predominance (55%). Over half of the participants (52%, *n* = 32) were identified as African American, reflecting the demographic diversity of the cohort. Diabetes mellitus was the leading cause of CKD, present in 93% (*n* = 58) of patients, while hypertension accounted for only 3% (*n* = 2). Other etiologies represented the remaining 3% of cases. The majority of patients (69%, *n* = 43) were undergoing hemodialysis, whereas 31% (*n* = 19) were on peritoneal dialysis, with a mean dialysis duration of 24 months. This distribution highlights the predominance of diabetes-related CKD in this population and the varied dialysis modalities employed.

**Table 1:** Demographic, clinical, and analytical characteristics of the patients.

Variable	Mean or percentage	SD	*p*-value *(vs CP) ** (vs EAT)
Clinical characteristics			
Age (years)	43.4	11	>0.05*
Gender:women (%)	55	–	>0.05*
Smoking habit (%)	59	–	>0.05*
Body mass index (kg/m2)	29	7	<0.03*
Systolic blood pressure (mmHg)	149	26	>0.05*
Diastolic blood pressure (mmHg)	82	14	>0.05*
Dialysis Vintage (months)	24	24	>0.05*
Biochemical results			
Hemoglobin (g/dL)	12	1	>0.05*
Hematocrit (%)	36	4	>0.05*
Fasting glucose (mg/dL)	183	96	>0.05*
Creatinine (mg/dL)	7	3	>0.05*
Total cholesterol (mg/dL)	163	34	>0.05*
HDL-C (mg/dL)	42	13	>0.05*
LDL-C (mg/dL)	95	37	>0.05*
Triglycerides (mg/dL)	151	110	>0.05*
Calcium (mg/dL)	9	1	>0.05*
Phosphate (mg/dL)	5	1	>0.05*
Ca × P product	47	13	>0.05*
Parathyroid hormone (pg/mL)	278	164	>0.05*
Alkaline phosphatase (mg/dL)	124	103	>0.05*
Albumin (mg/dL)	3	0.4	>0.05*
Uric acid (mg/dL)	5	1	>0.05*
CP (ng/mL)	4	5	>0.05*
Imaging			
Epicardial adipose tissue (mL)	96	67	<0.03*
Coronary artery calcium (HU)	369	997	<0.01**

Continuous variables were expressed as mean and standard deviation. Categorical variables were expressed as percentages (%). HDL-C, high-density lipoprotein cholesterol; LDL-C, low-density lipoprotein cholesterol; CP, C-peptide; EAT, epicardial adipose tissue; CAC, Coronary artery calcium. Bold means statistically significant. Single asterisk * means ´Given variable vs CP (C-Peptide). Double asterisk ** means ´Given variable vs EAT (Epicardial Adipose Tissue).

### CP levels and correlations

No significant difference in CP levels was observed between hemodialysis and peritoneal dialysis patients, nor correlation with any demographic or analytical parameters. However, a notable linear relationship emerged between body mass index (BMI) and CP levels. Patients with a higher BMI (mean 29 ± 7 kg/m^2^) exhibited elevated serum CP concentrations (mean 4 ± 5 ng/mL), with a statistically significant correlation (*r* = 0.30, *p* = 0.03). This suggests that adiposity may influence CP metabolism in CKD patients, independent of dialysis modality.

### EAT volume and CAC

**Figure 2:**
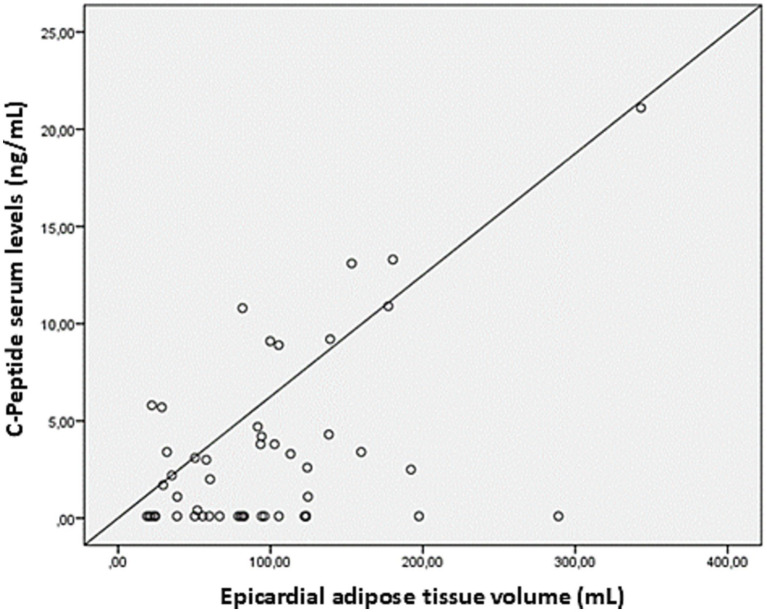
Linear correlation between serum levels of C-peptide and the volumes of epicardial adipose tissue. Scatter plot showing the relationship between epicardial adipose tissue volume (mL) and C-peptide serum levels (ng/mL). Each point represents an individual measurement, with the diagonal line indicating the reference for linear correlation.

**Figure 3:**
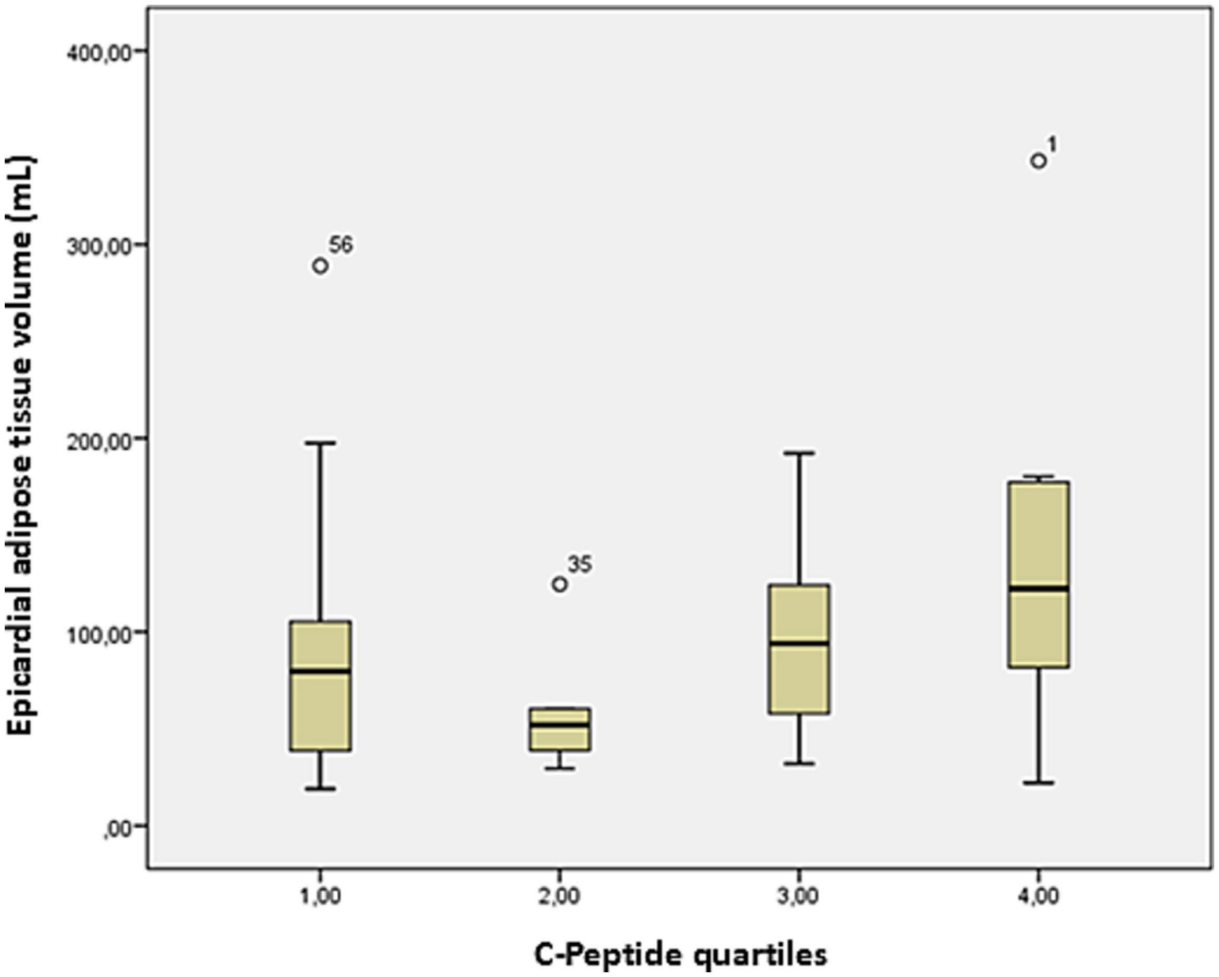
Box plot of EAT volume on cardiac CT and CP quartiles. Bars represent the minimum and maximum ranges of EAT volume values. The bottom and upper box limits encompass the 25–75% interquartile ranges. The black crossbars within the boxes represent median values. EAT volume tends to increase from lower to higher C-peptide quartiles, with the highest median values observed in quartile 4.

The average EAT volume was 96 ± 67 mL, while CAC scores were highly variable (369 ± 997). A strong positive association was observed between serum CP levels and EAT volume (*r* = 0.51, *p* = 0.03), reinforcing the potential role of metabolic factors in ectopic fat deposition (Figure 2). Stratification of EAT volume by CP quartiles showed a progressive increase, further underscoring this relationship (Figure 3). Additionally, EAT volume significantly correlated with CAC (*p* < 0.01), suggesting a link between pericardial fat and vascular calcification.

### CP and CAC relationship

Despite the association between EAT and CAC, no direct correlation was found between serum CP levels and CAC scores. This indicates that, although CP may contribute to EAT accumulation, its influence on coronary calcification may be indirect or mediated by other metabolic pathways. These results highlight the complex interplay between adiposity, metabolic markers, and cardiovascular risk among patients with CKD.

## Discussion

Our main finding was a significant correlation between CP and EAT volume in patients with T2DM receiving RRT. Additionally, as shown previously, EAT and CAC were strongly correlated. However, no association was found between CP serum level and CAC.

As expected due to decreased renal clearance, the mean circulating CP value in our sample (4 ± 5 ng/mL) was higher than the range of values previously reported in healthy people (0.36–3.61 ng/mL) (31) and in patients with T2DM at milder stages of CKD (~0.6–3 ng/mL) (32). The CP concentrations in our study were also within the range priorly reported by Guthoff et al. (33). In a population of 107 patients with ESKD awaiting kidney transplantation (4.47–8.28 ng/mL), who showed values significantly higher than a gender, age and BMI-matched cohort of healthy controls.

CP is sometimes used as a marker of insulin reserve to evaluate insulin needs in T2DM patients. Although insulin is routinely indicated in many dialysis units for glycemic control in patients with diabetes, CP is not included as part of their regular monitoring (33, 34). Despite its well-known hypoglycemic action, insulin has definite lipogenic effects that may be counterproductive. Besides, management of this drug in patients on dialysis is particularly challenging due to the diverse alterations in glucose and insulin metabolism that go beyond the reduced insulin clearance by the diseased kidneys (35). These alterations include reduced insulin secretion and sensitivity, the hypoglycemic effect of dialysis, decline in renal gluconeogenesis and deficient catecholamine release (36–38). Thus, CP monitoring may help gauge insulin needs and dosing in patients affected by ESKD.

Insulin promotes lipogenesis and suppresses hepatic gluconeogenesis. This occurs via the increase of glucose uptake in the adipocyte and the activation of lipogenic and glycolytic enzymes via covalent modification (39). Paradoxically, the positive effect of insulin on lipid production in the liver is maintained under conditions of insulin resistance, unlike its defective action on gluconeogenesis. Hence, the deleterious effects of hyperinsulinemia are related to increased cardiovascular risk, insulin resistance, weight gain, and an increase in visceral adipose tissue (33, 34).

Thus, insulin dosage adjustments should be made to improve the undesirable actions of this hormone. Dose reduction could be obtained with the introduction of new hypoglycemic drugs, such as glucagon-like peptide-1 receptor agonists (GLP1-RA) or dual GLP-1 and GIP (glucose-dependent insulinotropic polypeptide) receptor agonists. Not only do these drugs control glycemia in T2DM, but they also slow kidney dysfunction progression in patients with CKD not on dialysis (40–45), besides inducing significant weight loss without provoking episodes of hypoglycemia (46). Additionally, elimination of GLP-1RA is not dependent on renal function, representing an attractive option in dialysis (47).

As mentioned earlier, CP is a widely accepted biomarker of both *β*-cell function and insulin resistance (48). Insulin resistance, measured as the product of serum insulin by glucose level, has proven to be an adverse prognostic indicator of cardiovascular mortality (49). However, there is some evidence that CP levels are a better predictor of cardiovascular disease and overall mortality than serum insulin levels, while also supporting the increased mortality risk associated with an insulin-resistant state (50). This could also be due to CP having certain influence on the atherogenic process through the accumulation of oxidized LDL and the proliferation of aortic smooth muscle cells (16, 18, 51).

The role of CP in diabetic vascular complications is contradictory, as the progression of cardiovascular disease may be accelerated by insulin resistance mechanisms or the pathological functions of CP previously mentioned in the introduction. The statistically significant association found between this peptide and EAT aligns with the results found by Erman et al. (52). In people with metabolic syndrome, and may suggest a potential involvement of CP in the cardiovascular complications frequently present in patients with ESKD. Given that our study was a cross-sectional analysis, we were unable to determine whether CP is associated with further cardiovascular events or overall mortality in this population. Prospective studies with long-term follow-up are required to clarify whether CP is a predictor for cardiovascular endpoints in dialysis-dependent populations. EAT has been associated with cardiovascular risk and mortality in patients receiving dialysis (21, 23, 53, 54). In our prior publication, we reported that EAT was an independent factor for the prediction of inducible myocardial perfusion defects and higher CAC scores in ESKD (19). The physiological role of EAT is multifaceted, involving mechanical, metabolic, thermogenic, and endocrine/paracrine functions (55). Direct interaction between the inflamed EAT and the coronary arteries is believed to promote the development of atherosclerosis via the action of adipocytokines with pro-inflammatory properties. This process could be augmented by CP, which stimulates the release of chemokines from the adipocytes in EAT (12).

The CAC scores showed a highly skewed distribution with large variability (369 ± 997). Given our modest sample size, outlier exclusion was not applied. Unlike previous authors who applied logarithmic transformation of Agatston scores (56, 57), we opted to use a non-parametric statistical test (Spearman’s correlation) to assess the strength of the relationship between CAC and CP, as this test does not assume normality and is appropriate for skewed data. Nonetheless, the lack of correlation between CP levels and the extent of CAC may have been due to a power issue or the selection of relatively healthy and younger subjects for kidney transplantation.

Our study presents a few limitations besides its small size: it was a cross-sectional analysis; several patients were excluded due to missing serum CP values; the lack of regression analyses and adjustment for potential confounders (age, BMI, sex, diabetes duration, dialysis vintage) limits the independence of the associations found; no logarithmic transformation or sensitivity analysis was performed on the highly variable CAC scores; finally, no information on insulin type and dosage was obtained for most patients.

## Conclusion

In patients with T2DM on dialysis, a significant association between CP levels and EAT volume was found, highlighting a potential role of CP in the increased cardiometabolic risk characteristic of this population. Although a strong correlation between EAT and CAC was shown, no association between CAC and CP was reported. Longstanding observational prospective studies on larger samples and broader dialysis populations are required to assess the specific involvement of CP as a cardiovascular risk marker in patients with ESKD and as a predictor of CAC changes over time.

## Data Availability

The raw data supporting the conclusions of this article will be made available by the authors, without undue reservation.
